# Understanding the role of serotonin in psychiatric diseases

**DOI:** 10.12688/f1000research.10094.1

**Published:** 2017-02-23

**Authors:** Donatella Marazziti

**Affiliations:** 1 Dipartimento di Medicina Clinica e Sperimentale, Section of Psychiatry, University of Pisa, Via Roma, 67, 56100 Pisa, Italy

**Keywords:** psychiatric disorders, serotonin, pathophysiology, depression

## Abstract

Serotonin (5-HT) continues to attract researchers’ interest after almost a century. However, despite these efforts, its role has not yet been fully elucidated. It is now evident that 5-HT does not modulate single functions but rather a multiplicity of activities and behaviors present in both normal and several pathological conditions in a less deterministic way than previously assumed. This article aims to briefly review some of the latest advancements in the general role of 5-HT in psychiatry, particularly in depression, and offer the author’s personal reflections.

## Introduction

Serotonin (5-HT) is really puzzling. Originally discovered in the blood and in enthero-cromaffin cells and first thought to be a vasoconstrictor agent, 5-HT was also described after about twenty years in the central nervous system (CNS) ^1^, where it is now considered to represent one of the most diffuse, most influential, and probably most investigated neurotransmitters. However, in spite of decades of research all over the world, its nature remains elusive and its role is still surrounded by mystery.

Undoubtedly, the amount of data relative to 5-HT has permitted investigators to describe several aspects of its distribution, physiology, receptor subtypes, mode of functioning, and modulation of different activities in both the CNS and the periphery, including appetite, sleep, mood, sexuality, aggression/impulsivity, biological rhythms, motor control, memory, learning, neuronal degeneration, and gastrointestinal motility and vasoconstriction ^2– 5^. In addition, a rich literature shows the involvement of 5-HT in a series of disorders, including almost every neuropsychiatric domain. Indeed, all psychiatric disorders seem to be related to 5-HT dysfunctions, and many, if not all, psychotropic drugs used to treat psychopathological conditions interfere more or less directly with the 5-HT system ^6, 7^. However, as we analyze the evolution of hypotheses related to the involvement of 5-HT in the pathophysiology of psychiatric disorders, it is evident that the original enthusiasm based on classic theories, which now appears quite simplistic, has dampened.

## Discussion

The investigation of the neurobiology of depression is a clear example of this process and how old theories should be re-conceptualized on the basis of the latest findings. In the 1970s, depression was believed to be due to a deficit of 5-HT neurotransmission ^8– 10^. Taken together, convergent data from studies on tryptophan depletion, cerebrospinal fluid levels of 5-hydroxyindolacetic acid, neuroendocrine challenges, autopsy, and peripheral models of presynaptic serotonergic neurons, like platelets, seemed to support the presence of a reduced functioning of the 5-HT system in depression ^8– 11^. Interestingly, if we critically analyze those findings, it is evident that negative results were also present since the beginning but were mostly neglected. Moreover, attempts were made to encompass them in the defect hypothesis through rather complicated explanations, sometimes involving the 5-HT transporter (SERT) and one or more of the 5-HT receptor subtypes (of which there are now 14) discovered and characterized throughout the years ^12– 14^. Similarly, the function of the genes or genetic polymorphisms that have been continually proposed in depression in the last two decades was not always confirmed subsequently and led to less conclusive or inconclusive hypotheses ^15, 16^. In any case, all of these activities have promoted the synthesis and introduction into clinical practice of selective 5-HT re-uptake inhibitors (SSRIs), which represent one of the most successful psychopharmacological advancements and are still among the most widely prescribed drugs ^17^.

Currently, both clinicians and investigators realize the shortcomings of the previous findings and developments, and new data are considered more realistic but are taken with more caution.

First, there is clear-cut evidence of serotonergic dysfunctions in different psychopathological disorders (depression, anxiety disorders, eating disorders, schizophrenia, impulse control disorders, autism, and aggressive behaviors, just to mention the main ones), and, as just noted, several drugs with main activities on 5-HT, such as SSRIs, are reasonably effective therapeutic agents in all of these conditions. There has been a significant advancement not only in SSRIs but also in the field of psychosis, in which the development of second-generation antipsychotics targets specific 5-HT receptor subtypes ^17^. Unfortunately, the 5-HT dysfunctions widely described with different tools are not detected in all patients, and the non-response rate to serotonergic drugs is still quite significant (around 50% of the cases), and only 30% reach effective remission ^18^. Furthermore, no real achievement has been made in terms of possible predictors of response or prompt identification of individuals more prone to relapses in all the conditions related to 5-HT neurotransmission. However, more recent research has suggested changes in central 5-HT1B receptor binding, and the associated peripherally available biomarker p11 has been shown to be associated with response to both SSRIs and cognitive behavioral treatment of depression ^19^.

Second, it is clear that the level of “core” serotonergic dysfunction in the disorders where it has been described (or supposed to be present) should be considered still unknown, as it might result from one or more different processes, such as defective synthesis, release, re-uptake, catabolism, or metabolism of 5-HT per se, or from aberrations in one or more of the 14 receptors ^10, 11^.

Third, if (as suggested by some authors) decreased 5-HT functioning played a minimal or no role in depression ^20^ or could be even hyperfunctioning ^21^, it should be questioned whether 5-HT represents just one of the final, and not the main, factors in the neurological chain of events underlying those psychopathological symptoms attributable to this neurotransmitter ^22^.

It is not our intention to disregard the efforts and incredible achievements in the field of 5-HT that have inspired generations of researchers and continue to enliven them everywhere. However, we have to recognize that, without a doubt, the impact of the 5-HT hypothesis on the pathophysiology of psychiatric disorders has been quite robust, a sort of paradigm, according to Kuhn’s theory that may be in need of an update, including newer data suggesting 5-HT to be not *the one* but rather *one of many* important parts of the CNS that are involved in the pathophysiology of depression. We cannot exclude *tout court* that 5-HT is involved in the depressive psychopathology (for example, 30% of depressed patients reach remission from SSRI treatment) or exempt ourselves from exploring other working models ^21^. According to the recently emerging role of 5-HT in brain development, it is suggested that early alterations of this process, following environmental stressors or genetic liability, impair brain circuits, pathways, and differentiation and constitute a sort of basic “vulnerability” toward a greater risk of developing psychopathology ^16^. In this case, subsequent life events should act through epigenetic mechanisms acting on stress response and emotion regulation ^23, 24^. Of interest, both SERT-s allele carriers and sensory processing sensitivity are associated with greater sensitivity to environmental stimuli in humans ^25^. Long-follow-up studies and impact of stressors in childhood and adolescence, together with studies on human DNA methylation or acetylation, should be planned to explore epigenetic mechanisms more thoroughly. It would also be interesting to ascertain whether different types of stressors (familial, emotional, and environmental) should produce different biochemical effects on the 5-HT system, why some individuals become ill and others do not, and what the individual factors promoting resilience are.

Other recent biological hypotheses on the role of 5-HT in depression (and perhaps in all other disorders where serotonergic alterations have been detected) highlight how this neurotransmitter is part of a more complex network including even the immune system and the whole body ^26, 27^ or might play a more general role in the energy homeostasis through modulation of mitochondria activity ^21^.

In any case, all research in the 5-HT field might benefit from a deeper knowledge of more precise anatomical data in humans. Undoubtedly, the latest functional magnetic resonance imaging approaches linking brain circuits to SERT-gene polymorphism, emotional processing, and pharmacological challenges appear extremely helpful and promising in this sense ^28– 30^.

## Conclusions

The precise role of 5-HT in psychiatric disorders remains elusive after decades of intensive research. Currently, two main notions are widely accepted in this field. One is that the serotonergic dysfunctions cannot be related to distinct nosological entities but rather to symptoms/dimensions shared by different conditions ^31^. The second, related to the first, is that the 5-HT hypothesis of psychopathology has become less casual and tends to be more comprehensive, albeit cautious. Therefore, currently, different elements are taken into account when considering the role of 5-HT: its relationships with other neurotransmitters, neuropeptides, and neurotrophins ^10^ and how it may regulate emotions, cognition, motivation, and behaviors to produce different clinical pictures according to individual vulnerability due to genetic load, life events, and environmental stressors ^32^.

In 1998, John Greden had already written about 5-HT that “much we have learned” but that there is “So much to discover” ^33^. After twenty years, we are strongly convinced that “the best is yet to come”, again to quote Greden ^33^. That is, 5-HT continues (and probably will continue for a long time) to represent a “hot” topic in neuropsychiatry, a real challenge for research, and a “never-ending story” ^34, 35^ that hopefully will permit us to disentangle one of the most fascinating mysteries of our nature and lead to really innovative pharmacological and psychosocial interventions effective in a broad range of psychiatric disorders.

## Abbreviations

5-HT, serotonin; CNS, central nervous system;SERT, serotonin transporter; SSRI, selective serotonin re-uptake inhibitor.
